# Verification of grip strength as an evaluation tool for locomotive syndrome in rheumatoid arthritis

**DOI:** 10.1016/j.afos.2024.07.001

**Published:** 2024-07-25

**Authors:** Yasumori Sobue, Mochihito Suzuki, Yoshifumi Ohashi, Ryo Sato, Hironobu Kosugiyama, Yusuke Ohno, Junya Hasegawa, Takaya Sugiura, Kenya Terabe, Shuji Asai, Shiro Imagama

**Affiliations:** a Department of Orthopedic Surgery, Japanese Red Cross Aichi Medical Center Nagoya Daiichi Hospital, 3-35 Michishita, Nakamura, Nagoya, Aichi, 453-8511, Japan; b Department of Orthopedic Surgery and Rheumatology, Nagoya University Graduate School of Medicine, 65 Tsurumai, Showa, Nagoya, Aichi, 466-8550, Japan; c Department of Orthopedic Surgery, Japan Community Healthcare Organization Kani Tono Hospital, 1221-5 Dota, Kani, Gifu, 509-0206, Japan; d Department of Orthopedic Surgery, Aichi Medical University, Graduate School of Medicine, 1-1 Karimata yazako, Nagakute, Aichi, 480-1195, Japan; e Department of Orthopedic Surgery, Yokkaichi Municipal Hospital, 2-2-37 Shibata, Yokkaichi, Mie, 510-8567, Japan

**Keywords:** Grip strength, Locomotive syndrome, Rheumatoid arthritis, Sarcopenia, 25-Question geriatric locomotive function scale

## Abstract

**Objectives:**

Locomotive syndrome (LS) leads to reduced physical function and a high risk of becoming bedridden. Grip strength serves as an indicator of upper limb and overall physical function. Rheumatoid arthritis (RA) patients with reduced grip strength frequently show finger and wrist joint inflammation. The purpose of this study was to verify grip strength as an evaluation tool for physical function and LS in RA patients.

**Methods:**

As part of an ongoing multicenter observational study, 591 consecutive RA patients whose background information was available, including data for the 25-question Geriatric Locomotive Function Scale (GLFS-25) and grip strength, were examined. LS was defined as a GLFS-25 score ≥ 16 points. Finger and wrist joint inflammation were defined as tender or swollen joints.

**Results:**

Among the 591 patients, 244 (41.3%) patients had LS, and 167 (28.3%) were male. Receiver operating characteristic curve analysis yielded cut-off values of grip strength for LS of 24 kg (specificity 72.2%; sensitivity 62.7%) for males and 17 kg (specificity 65.7%; sensitivity 67.6%) for females. Multivariable logistic regression analysis revealed a significant association of grip strength with LS, even after adjusting for finger and wrist joint inflammation.

**Conclusions:**

LS was significantly associated with grip strength, even after adjusting for the presence of finger and wrist joint inflammation. We recommend adopting grip strength measurement as a screening tool for evaluating LS and guiding interventions.

## 1 Introduction

Life expectancy is projected to increase globally, and extended lifespan will increase the global burden of late-life diseases [1]. Preserving and enhancing physical function is important from the perspectives of preventing the bedridden state and increasing the quality of life (QoL) in an aging population. As a society with a super-aging population and a very low birth rate, Japan must face the challenge of supporting active aging [2]. Locomotive syndrome (LS), introduced by the Japanese Orthopedic Association, describes a decline in movement ability due to musculoskeletal issues. It is a critical condition that often necessitates caregiving, as it can lead to a bedridden state [2,3], and influences QoL and healthy life expectancy [4].

Rheumatoid arthritis (RA) is a chronic autoimmune disease marked by systemic inflammation and joint destruction, leading to physical impairments. Tumor necrosis factor-alpha and other inflammatory cytokines produced in the process of RA pathogenesis have catabolic effects on skeletal muscle [5]. Consequently, individuals with RA are at an increased risk of developing musculoskeletal issues, including LS. Studies suggest a higher prevalence of LS among RA patients compared to the broader Japanese population across all age categories [6,7]. This is an important issue, especially for the growing population of older RA patients [8]. Identifying RA patients at risk of LS is crucial for orthopedic rheumatologists.

Grip strength, which can be easily assessed in a clinical environment, reflects not only upper limb function but also whole body physical function [9]. It has been reported that a low grip strength was associated with future LS and the progression of locomotive risk stage [10], as well as mortality [11]. Diminished grip strength is a factor in diagnosing sarcopenia [12]. However, RA patients often exhibit finger and wrist joint inflammation [13]. Patients with hand diseases reportedly demonstrated significantly weaker grip strength compared to those without [14]. Accordingly, this study sought to verify grip strength as an evaluation tool for LS in RA patients, with a focus on finger and wrist joint inflammation.

## 2 Methods

### 2.1 Patients

Between June and August 2021, a cohort of 630 consecutive RA patients visited the Japanese Red Cross Aichi Medical Center Nagoya Daiichi Hospital, Japan Community Health Care Organization Kani Tono Hospital, and Yokkaichi Municipal Hospital. This study was conducted as part of the Tsurumai - Frailty and Locomotive syndrome of rheumatoid Arthritis for Globalization [T-FLAG] study, an ongoing multicenter observational study designed to analyze frailty and LS in RA patients in clinical practice (start date: June 1, 2020). This study was approved by the Ethics Committees of Nagoya University School of Medicine (2017–0271), Japanese Red Cross Nagoya Daiichi Hospital (2020–451), Japan Community Health Care Organization Kani Tono Hospital (20110901), and Yokkaichi Municipal Hospital (2017–29). We disclosed information pertaining to the study at the cooperating facilities according to the procedure stipulated by the respective Ethics Committees. Informed consent was obtained from all patients. This study was conducted in accordance with the World Medical Association of Helsinki ethical principles for medical research involving human subjects. Patients’ individual information was anonymized.

Among these, data on clinical characteristics, including scores for the 25-question Geriatric Locomotive Function Scale (GLFS-25) [3], Clinical Disease Activity Index (CDAI), and grip strength, were available for 591 patients, all of whom fulfilled the 2010 American College of Rheumatology (ACR)/European League Against Rheumatism (EULAR) classification criteria [15]. The documented data encompassed a wide range of patient details, including age, duration of disease, sex, body mass index (BMI), Steinbrocker classification stage (evaluated at the most progressed joint), drug therapy (glucocorticoid [GC], MTX, other conventional synthetic DMARDs [csDMARDs] including salazosulfapyridine, tacrolimus, bucillamine, and iguratimod, and biological DMARDs [bDMARDs]/targeted synthetic DMARDs [tsDMARDs]), rheumatoid factor (RF), C-reactive protein (CRP), matrix metalloproteinase-3 (MMP-3), swollen 28-joint count (SJC), tender 28-joint count (TJC), subject's pain and global assessment of disease activity visual analog scale (VAS), physician's global assessment of disease activity VAS, Health Assessment Questionnaire-Disability Index (HAQ-DI), and grip strength of the dominant side (ie, side with the higher value).

### 2.2 Definition of LS

LS is identified when an individual scores ≥ 16 points on GLFS-25, which was specifically designed to facilitate the early identification of LS (Supplementary Table 1). This scale includes a total of 25 questions, segmented into categories: four questions about “Body pain” experienced over the past month (questions 1 through 4) and 21 questions about “Movement-related difficulty” (questions 5 through 7), “Usual care” (questions 8 through 11, and 14), “GLFS-5” (questions 12, 13, 15, 17, and 20), “Social activities” (questions 18, 21, 22, and 23), and “Cognitive” (questions 24 and 25) over the past month [3]. Answers are given on a scale from 0 (no impairment) to 4 (severe impairment) points, allowing for a cumulative score ranging from 0 (no symptoms) to 100 (most severe) points. The severity of LS is categorized based on the GLFS-25 scores: GLFS-25 < 7 points correspond to Stage 0; 7–15 points correspond to Stage 1; 16–23 points correspond to Stage 2 (ie, LS), and ≥ 24 points correspond to Stage 3. Stage 1 indicates the onset of reduced mobility, whereas Stages 2 and 3 represent a further decline, with Stage 3 affecting social participation significantly [3].

### 2.3 Grip strength, and finger and wrist joint inflammation

Grip strength was assessed using Smedley Spring handgrip dynamometers (TTM Smedley Dynamo Meter; Tsutsumi, Tokyo, Japan) while participants stood with their elbows fully extended. Each participant's right and left hands were tested twice, and the highest measurement was selected for the analysis in line with recommendations from the Asian Working Group for Sarcopenia [12].

Finger and wrist joint inflammation were characterized by tenderness and/or swelling in the wrist, metacarpophalangeal, and proximal interphalangeal joints of the hand on the dominant side. The side considered dominant was determined by which hand had the higher value of grip strength. The terms “tender” and “swollen” joints describe the presence of pain on touch and visible enlargement in and around the joints, respectively [16].

### 2.4 Missing data

The distribution of unavailable data (final numbers and percentages in parentheses) is detailed as follows: 5 for the duration of disease (586/591, 99.2%), 15 for BMI (576/591, 97.5%), 11 for Steinbrocker stage (580/591, 98.1%), 18 for RF (573/591, 97.0%), 19 for MMP3 (572/591, 96.8%), and 1 for CRP (590/591, 99.8%).

### 2.5 Statistical analysis

Continuous variables were presented as the mean with their standard deviation (SD) and analyzed using the unpaired *t*-test. Ordinal and categorical variables are shown as percentage and examined through Fisher's exact test. Correlations between GLFS-25 and grip strength were analyzed using Spearman's rank correlation coefficients. Receiver operating characteristic (ROC) curves were generated to assess the association between LS and grip strength, with the best cut-off point determined by the location nearest to the upper left corner of the curve. A multivariable logistic regression analysis was conducted to assess the independent impact of each factor on LS, incorporating two models. Model 1 included age, disease duration, sex, Steinbrocker stage, use of GC, MTX, other csDMARD, bDMARD/tsDMARD, finger and wrist joint inflammation, and grip strength, based on previously reported LS-related factors [17]. Due to significant correlations found between grip strength and HAQ-DI, as well as between finger and wrist joint inflammation and disease activity measures [18], both HAQ-DI and disease activity were excluded from Model 1, applying to the overall analysis as well as separately for males and females. Model 2 adjusted for HAQ-DI and disease activity (CDAI) measures instead of grip strength and finger and wrist joint inflammation, providing a complementary perspective on the factors influencing LS in RA patients.

Statistical analyses were performed with EZR (Saitama Medical Center, Jichi Medical University, Saitama, Japan; http://www.jichi.ac.jp/saitama-sct/SaitamaHP.files/statmed.html), a graphical user interface for R (The R Foundation for Statistical Computing, Vienna, Austria) [19]. A P-value of less than 0.05 was deemed to indicate statistical significance.

## 3 Results

### 3.1 *Patient characteristics*

Patient characteristics are summarized in Table 1 (total, LS, and non-LS) and Supplementary Table 2 (male and female). Overall, mean age of the 591 patients (167 males, 28.3%) was 67.4 years, disease duration was 11.8 years, CDAI was 6.5, GLFS-25 score was 19.7 points, and grip strength was 20.1 kg. There were 244 (41.3%) patients with LS. Mean age, disease duration, CDAI, and proportions of patients with GC use, b/tsDMARD use, and finger and wrist joint inflammation were significantly higher, and the proportion of patients with MTX use and grip strength were significantly lower, in patients with LS than in those without (ie, non-LS). Mean disease duration and the proportion of patients with MTX use were significantly lower in males than in females. Mean grip strength was 27.2 kg in males and 17.4 in females. Mean grip strength in males was significantly lower in patients with finger and wrist joint inflammation (24.6 kg) than in those without (28.4 kg), and grip strength in females was significantly lower in patients with finger and wrist joint inflammation (15.7 kg) than in those without (18.3 kg) (Table 2). Additionally, mean CRP, SJC, TJC, VAS, CDAI, HAQ-DI, and GLFS-25 values were significantly higher in patients with finger and wrist joint inflammation than in those without.

**Table 1:** Demographics and clinical characteristics of patients.

Variables		Total (N = 591)	Non-LS (N = 347)	LS (N = 244)	P-value
Age, yrs	Mean (SD)	67.4 (13.9)	64.9 (13.8)	70.9 (13.4)	<0.001
Duration of disease, yrs	Mean (SD)	11.8 (9.8)	10.7 (9.0)	13.4 (10.7)	0.001
Sex, female, %		71.7	68.9	75.8	0.078
BMI, kg/m^2^	Mean (SD)	22.0 (3.8)	21.8 (3.6)	22.2 (4.0)	0.242
Steinbrocker stage (1/2/3/4), %		38.4/25.0/15.2/21.4	43.8/25.0/15.9/15.3	30.8/25.0/14.2/30.0	<0.001
Glucocorticoid use, %		31.5	25.4	40.2	<0.001
Methotrexate use, %		59.6	65.7	50.8	<0.001
Other csDMARD use, %		44.5	39.2	52	0.002
bDMARD or tsDMARD use, %		37.4	33.7	42.6	0.031
Rheumatoid factor positive, %		70.9	68.9	73.6	0.262
CRP, mg/dL	Mean (SD)	0.4 (1.1)	0.3 (0.4)	0.7 (1.6)	<0.001
MMP-3, ng/mL	Mean (SD)	98.0 (101.3)	76.5 (72.7)	128.7 (125.6)	<0.001
Swollen joint count	Mean (SD)	0.7 (1.9)	0.5 (1.4)	1.0 (2.4)	0.002
Tender joint count	Mean (SD)	2.0 (3.8)	1.0 (2.2)	3.5 (5.0)	<0.001
Subject's assessment of pain VAS, mm	Mean (SD)	20.3 (23.5)	10.8 (15.8)	33.8 (25.9)	<0.001
Subject's global assessment of disease activity VAS, mm	Mean (SD)	20.6 (23.2)	10.9 (15.8)	34.4 (24.9)	<0.001
Physician's global assessment of disease activity VAS, mm	Mean (SD)	17.3 (20.6)	9.0 (13.7)	29.1 (22.9)	<0.001
CDAI	Mean (SD)	6.5 (7.5)	3.5 (4.6)	10.8 (8.7)	<0.001
HAQ-DI	Mean (SD)	0.50 (0.72)	0.10 (0.20)	1.05 (0.83)	<0.001
GLFS-25	Mean (SD)	19.7 (19.9)	7.3 (4.4)	37.3 (20.1)	<0.001
Dominant side (right/left/equal), %		47.4/35.2/17.4	48.7/35.2/16.1	45.5/35.2/19.3	0.569
Grip strength, kg	Mean (SD)	20.1 (9.5)	23.1 (8.9)	15.9 (8.8)	<0.001
Finger and wrist joint inflammation, %		34.3	27.1	44.7	<0.001

Locomotive syndrome (LS), 25-question Geriatric Locomotive Function Scale (GLFS-25) ≥ 16 points; BMI, body mass index; Other csDMARD, conventional synthetic disease-modifying antirheumatic drug (DMARD) including salazosulfapyridine, tacrolimus, bucillamine, and iguratimod; bDMARD, biological DMARD; tsDMARD, targeted synthetic DMARD; CRP, C-reactive protein; MMP-3, matrix metalloproteinase-3; VAS, visual analog scale; CDAI, clinical disease activity index; HAQ-DI, health assessment questionnaire-sisability index; Dominant side, side with the higher value of grip strength; SD, standard deviation. P < 0.05 was considered statistically significant.

**Table 2:** Demographics and clinical characteristics of patients categorized by finger and wrist joint inflammation.

Variables		Without finger and wrist joint inflammation (N = 388)	With finger and wrist joint inflammation (N = 203)	P-value
Age, yrs	Mean (SD)	67.6 (14.2)	67.0 (13.4)	0.589
Duration of disease, yrs	Mean (SD)	12.1 (10.1)	11.2 (9.3)	0.318
Sex, female, %		70.6	73.9	0.442
BMI, kg/m^2^	Mean (SD)	22.0 (3.8)	21.9 (3.8)	0.700
Steinbrocker stage (1/2/3/4), %		39.6/25.1/15.0/20.3	36.3/24.9/15.4/23.4	0.807
Glucocorticoid use, %		29.4	35.5	0.136
Methotrexate use, %		61.9	55.2	0.133
Other csDMARD use, %		43.8	45.8	0.664
bDMARD or tsDMARD use, %		33.2	45.3	0.004
Rheumatoid factor positive, %		67.6	77.0	0.021
CRP, mg/dL	Mean (SD)	0.4 (0.7)	0.6 (1.6)	0.043
MMP-3, ng/mL	Mean (SD)	95.0 (92.9)	103.6 (115.4)	0.334
Swollen joint count	Mean (SD)	0.1 (0.5)	1.7 (2.9)	<0.001
Tender joint count	Mean (SD)	0.6 (1.2)	4.8 (5.3)	<0.001
Subject's assessment of pain VAS, mm	Mean (SD)	14.5 (20.0)	31.5 (25.5)	<0.001
Subject's global assessment of disease activity VAS, mm	Mean (SD)	14.8 (19.9)	31.9 (24.7)	<0.001
Physician's global assessment of disease activity VAS, mm	Mean (SD)	12.3 (17.8)	26.9 (22.1)	<0.001
CDAI	Mean (SD)	3.4 (4.5)	12.4 (8.6)	<0.001
HAQ-DI	Mean (SD)	0.38 (0.65)	0.71 (0.81)	<0.001
GLFS-25	Mean (SD)	16.4 (17.0)	25.8 (23.4)	<0.001
Dominant side (right/left/equal), %		47.9/38.1/13.9	46.3/29.6/24.1	0.005
Grip strength, kg	Mean (SD)	21.3 (9.6)	18.0 (9.1)	<0.001
**Male**		N = 114	N = 53	
Grip strength, kg	Mean (SD)	28.4 (10.3)	24.6 (10.6)	0.028
**Female**		N = 274	N = 150	
Grip strength, kg	Mean (SD)	18.3 (7.5)	15.7 (7.2)	0.001

See Table 1 for descriptions of abbreviations.

### 3.2 *Association between LS and grip strength*

**Fig. 1:**
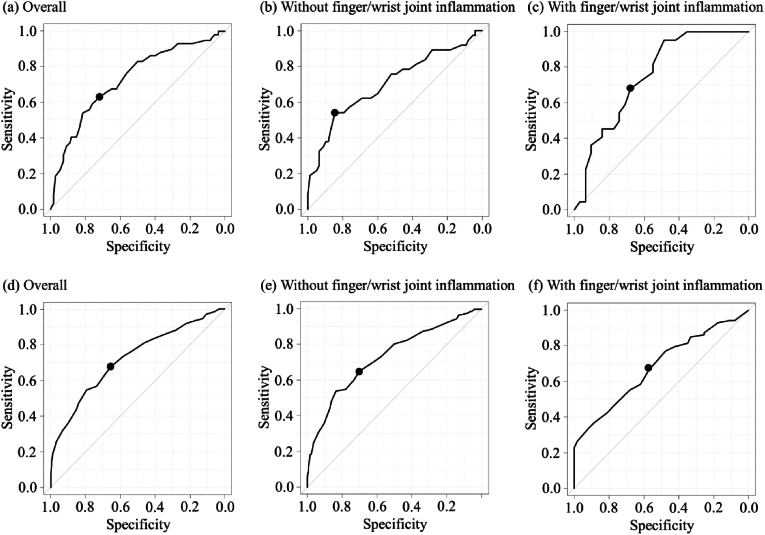
ROC curves for locomotive syndrome and grip strength in males: (a) overall, (b) without finger and wrist joint inflammation, (c) with finger and wrist joint inflammation; and females: (d) overall, (e) without finger and wrist joint inflammation, and (f) with finger and wrist joint inflammation.

**Fig. 2:**
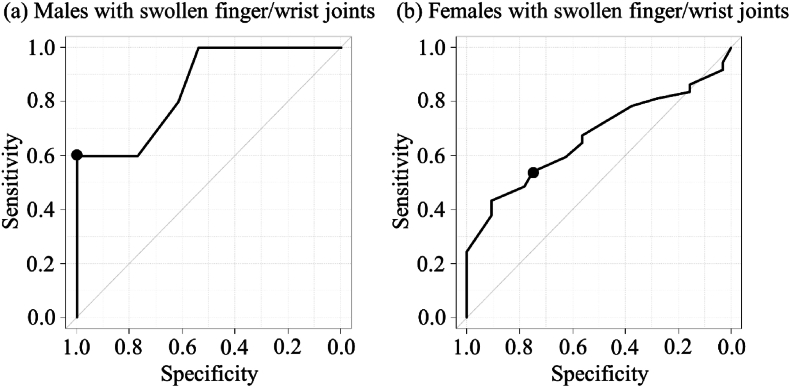
ROC curves for locomotive syndrome and grip strength in (a) males with swollen finger and wrist joints and (b) females with swollen finger and wrist joints.

In a scatter plot showing the relationship between GLFS-25 and grip strength, the correlation coefficient between the two variables was −0.469 (overall), −0.444 (male), −0.488 (female), −0.439 (without finger and wrist joint inflammation), and −0.466 (with finger and wrist joint inflammation) (P < 0.001). In ROC curve analyses, the cut-off value of grip strength for LS was 24 kg (specificity 72.2%; sensitivity 62.7%; area under the curve [AUC] 0.727 [95% confidence interval [CI]: 0.646–0.809]) in males (Fig. 1a) and 17 kg (specificity 65.7%; sensitivity 67.6%; AUC 0.723 [95%CI: 0.674–0.772]) in females (Fig. 1d). According to the presence or absence of finger and wrist joint inflammation, the cut-off value of grip strength for LS was 21 kg in males without finger and wrist joint inflammation (specificity 84.4%; sensitivity 54.1%) (Figs. 1b), 23 kg in males with finger and wrist joint inflammation (specificity 67.7%; sensitivity 68.2%) (Fig. 1c), 17 kg in females without finger and wrist joint inflammation (specificity 69.9%; sensitivity 65.3%) (Fig. 1e), and 16 kg in females with finger and wrist joint inflammation (specificity 57.1%; sensitivity 66.7%) (Fig. 1f). Our analysis differentiated the effects of tender and swollen joints. While tender joints did not modify these initially established cut-off values for either gender, swollen joints led to increased specificity in the measurement, necessitating lower grip strength cut-off values. Specifically, for males, the grip strength cut-off was reduced to 15 kg (specificity 100%; sensitivity 60.0%; AUC 0.854 [95%CI: 0.650–1.000]) (Fig. 2a), and for females, it was adjusted to 13 kg (specificity 75.0%; sensitivity 54.1%; AUC 0.669 [95%CI: 0.540–0.798]) (Fig. 2b).

### 3.3 *Odds ratios of grip strength and each factor for LS*

Table 3 shows the odds ratios (ORs) for LS obtained in the logistic regression analyses. LS showed a significant association with grip strength overall and in both males and females (Model 1, overall, OR: 0.91 [95%CI: 0.89–0.94]); male, OR: 0.92 [95%CI: 0.88–0.96]); female, OR: 0.90 [95%CI: 0.86–0.93]), regardless of finger and wrist joint inflammation. In Model 2, LS showed a significant association with CDAI (OR: 1.10 [95%CI: 1.05–1.14]), and HAQ-DI (OR: 1.61 [95%CI: 1.46–1.77]) in the overall analysis.

**Table 3:** Odds ratios for LS by logistic regression analyses.

Variables	Overall (Model 1)			Overall (Model 2)			Male			Female		
	OR	(95% CI)	P-value	OR	(95% CI)	P-value	OR	(95% CI)	P-value	OR	(95% CI)	P-value
Age, yrs	1.02	(1.00–1.03)	0.034∗	1.03	(1.01–1.05)	<0.001∗	0.99	(0.95–1.02)	0.524	1.03	(1.01–1.05)	0.004∗
Duration of disease, yrs	1.00	(0.98–1.02)	0.902	0.98	(0.95–1.01)	0.209	1.03	(0.97–1.10)	0.310	0.99	(0.97–1.02)	0.530
Sex, female	0.77	(0.46–1.28)	0.312	1.40	(0.76–2.59)	0.278						
Steinbrocker stage (3/4)	0.83	(0.52–1.32)	0.427	0.67	(0.34–1.32)	0.247	0.76	(0.30–1.93)	0.567	0.74	(0.42–1.30)	0.299
Glucocorticoid use	1.56	(1.04–2.33)	0.030∗	1.51	(0.85–2.66)	0.159	1.77	(0.80–3.90)	0.156	1.42	(0.88–2.30)	0.152
Methotrexate use	0.90	(0.59–1.37)	0.630	1.33	(0.72–2.47)	0.362	0.88	(0.39–1.98)	0.750	1.05	(0.63–1.74)	0.853
Other csDMARD use	1.64	(1.11–2.43)	0.013∗	1.93	(1.09–3.40)	0.023∗	2.74	(1.22–6.16)	0.015∗	1.46	(0.91–2.33)	0.118
bDMARD or tsDMARD use	1.58	(1.04–2.39)	0.033∗	1.33	(0.73–2.44)	0.348	1.07	(0.48–2.42)	0.861	1.99	(1.19–3.33)	0.008∗
CDAI				1.10	(1.05–1.14)	<0.001∗						
Finger and wrist joint inflammation	1.75	(1.19–2.59)	0.005∗				1.27	(0.57–2.81)	0.556	2.11	(1.32–3.36)	0.002∗
HAQ-DI				1.61	(1.46–1.77)	<0.001∗						
Grip strength, kg	0.91	(0.89–0.94)	<0.001∗				0.92	(0.88–0.96)	<0.001∗	0.90	(0.86–0.93)	0.001∗

OR, odds ratio; CI, confidence interval; See Table 1 for descriptions of other abbreviations; Odds ratio for 0.1 point increase in HAQ-DI; ∗P < 0.05 was considered statistically significant.

## 4 Discussion

The present study is the first to investigate the association between grip strength and LS in RA patients, paying particular attention to finger and wrist joint inflammation, namely tender and swollen joints. The findings reveal a significant correlation between grip strength and LS, persisting despite adjustments for age, the duration of disease, RA medication and the level of disease activity (ie, previously reported factors of LS) [17,20,21], and finger and wrist joint inflammation. Interestingly, significant associations with LS persisted even when grip strength was used as a substitute for HAQ-DI and finger and wrist joint inflammation replaced CDAI, highlighting grip strength and joint inflammation as particularly effective alternative measures in assessing LS. While the cut-off value of grip strength for LS in female patients (17 kg) was consistent with the criterion for diagnosing sarcopenia (ie, < 18 kg for females), that in male patients (24 kg) was lower than the criterion value (ie, < 28 kg for males) [12]. Furthermore, when the analysis was restricted to patients with swollen finger and wrist joints, these conditions led to increased specificity in measurements, requiring lower grip strength cut-off values (male: 15 kg, female: 13 kg). This underscores the need for clinicians to consider adjusted cut-off values for grip strength in RA patients with swollen joints to more accurately assess the risk of LS.

Reduced grip strength may result from sarcopenia, defined by the gradual and widespread reduction of skeletal muscle mass and strength [12]. Causes of muscle loss are multifactorial and include aging, disuse, malnutrition, and diseases (cachexia, inflammation diseases such as RA) [22]. In general, males have more muscle than females [22]. Focusing on sex-related differences in the nature and phenotype of muscles, a previous study reported physiological and pathological factors associated with muscle loss (ie, mass, force, energy metabolism, protein turnover, insulin responsiveness, mitochondrial presence, muscle repair, and myofilament cross bridge kinetics) [22]. However, given the multifactorial etiology of muscle loss, it is difficult to determine the significance of muscle loss caused by RA or to compare between male and female RA patients who do not share the same background. It has been noted that male RA patients generally experience a milder disease progression and better response to RA therapy than female patients [23]. Moreover, a longer duration of the disease has been linked to poorer grip strength [24]. While these observations remain speculative, they suggest that sex differences may influence the impact of RA on the reduction of grip strength and overall muscle strength. This implies that factors beyond the observed differences in disease progression and treatment responses might influence these outcomes. Therefore, considering sex differences is crucial when assessing the effects of RA on muscle degradation.

In patients with RA, factors such as age [25], the duration of disease [24], and the level of disease activity [26] can affect grip strength. In the present study, grip strength was significantly associated with LS even when accounting for these variables. While there is no doubt that limb muscle strength is very important when evaluating LS, measuring grip strength is notably simpler and quicker, taking approximately 5 s, compared to the few minutes required for assessing the strength and function of the lower limbs through tests such as the stand-up test and the two-step test. Previous research has indicated that early involvement of the hands in inflammatory arthritis was a strong indicator of unfavorable disease outcomes over the long term [27]. Additionally, in the present study, disease activity and physical function (HAQ-DI and GLFS-25 values) were significantly worse in patients with finger and wrist joint inflammation than in those without. This pattern suggests a link between finger and wrist joint inflammation and increased disease activity. Another study highlighted a significant correlation between finger and wrist joint inflammation and DAS28-CRP [18]. These reports suggest that monitoring grip strength and finger and wrist joint inflammation in RA patients may lead to detection of the worsening of disease activity, prevention of LS, and prevention of adverse health consequences. Engaging in appropriate physical activity can enhance both lower limb and grip strength [28]. From the standpoint of LS prevention, exercise is particularly beneficial when used alongside RA medication, offering a combined effect.

The study is subject to a few limitations. Firstly, it focused exclusively on Japanese patients with RA, with fewer male patients than female patients. To validate the obtained cut-off values, future research with a broader participant base will be required. Secondly, radiographic evaluations such as joint destruction and bone erosion were not performed. However, despite the absence of radiographic changes, the presence of tender and swollen joints alone can diminish grip strength. Thirdly, there was no collection of data on any upper limb surgeries that the participants have undergone, which could affect grip strength. Lastly, evaluations of locomotive functions, including the stand-up test and the two-step test, as well as tests for sarcopenia in the lower limbs, were not conducted as part of this study.

## 5 Conclusions

To summarize, our research explored the association between grip strength and LS in RA patients. We found a significant association between LS and grip strength, even after adjusting for age, the duration of disease, the level of disease activity, and finger and wrist joint inflammation. Notably, our analysis showed that swollen finger and wrist joints require lower grip strength cut-off values due to increased specificity in these measurements.

## Data availability statement

The data that support the findings of this study are available from the corresponding author upon reasonable request.

## Credit author statement

**Yasumori Sobue:** Conceptualization, Methodology, Software, Validation, Formal analysis, Investigation, Data curation, Visualization, Writing - Original draft preparation, Writing - Reviewing and Editing. **Mochihito Suzuki:** Conceptualization, Methodology, Software, Validation, Formal analysis, Investigation, Data curation, Visualization, Writing - Original draft preparation, Writing - Reviewing and Editing. **Yoshifumi Ohashi:** Conceptualization, Methodology, Software, Validation, Formal analysis, Investigation, Data curation, Visualization, Writing - Original draft preparation, Writing - Reviewing and Editing. **Ryo Sato:** Investigation, Data curation, Writing- Reviewing and Editing. **Hironobu Kosugiyama:** Investigation, Data curation, Writing- Reviewing and Editing. **Yusuke Ohno:** Investigation, Data curation, Writing - Reviewing and Editing. **Junya Hasegawa:** Investigation, Data curation, Writing - Reviewing and Editing. **Takaya Sugiura:** Investigation, Data curation, Writing - Reviewing and Editing. **Kenya Terabe:** Investigation, Data curation, Writing- Reviewing and Editing. **Shuji Asai:** Investigation, Data curation, Writing- Reviewing and Editing. **Shiro Imagama:** Supervision, Conceptualization, Methodology, Writing - Reviewing and Editing.

## Conflicts of interest

The authors declare no competing interests.

## Declaration of generative AI in scientific writing

During the preparation of this work the authors used ChatGPT, developed by OpenAI, for assistance with writing and spelling corrections. After using this tool, the authors reviewed and edited the content as needed and take full responsibility for the content of the publication.
