# Supplementary material for: Verification of grip strength as an evaluation tool for locomotive syndrome in rheumatoid arthritis
Source: Osteoporos Sarcopenia. 2024 Jul 25;10(3):119–25. doi: 10.1016/j.afos.2024.07.001 (PMC11471095; doi:10.1016/j.afos.2024.07.001)
Supplement: Multimedia component 1 [file mmc1.docx]

| **Supplementary Table 1.**  25-question Geriatric Locomotive Function Scale (GLFS-25). | | | | | |
| --- | --- | --- | --- | --- | --- |
| GLFS-25 items | 0 points | 1 point | 2 points | 3 points | 4 points |
| 1. Neck or upper limb pain | Not painful | Slightly painful | Moderately painful | Quite painful | Severely painful |
| 2. Back, lower back, or buttocks pain | Not painful | Slightly painful | Moderately painful | Quite painful | Severely painful |
| 3. Lower limb pain | Not painful | Slightly painful | Moderately painful | Quite painful | Severely painful |
| 4. Painful to move body in daily life | Not painful | Slightly painful | Moderately painful | Quite painful | Severely painful |
| 5. Difficult to get up from a bed or lie down | Not difficult | Slightly difficult | Moderately difficult | Quite difficult | Severely difficult |
| 6. Difficult to stand up from a chair | Not difficult | Slightly difficult | Moderately difficult | Quite difficult | Severely difficult |
| 7. Difficult to walk inside the house | Not difficult | Slightly difficult | Moderately difficult | Quite difficult | Severely difficult |
| 8. Difficult to put on and take off shirt | Not difficult | Slightly difficult | Moderately difficult | Quite difficult | Severely difficult |
| 9. Difficult to put on and take off trousers and pants | Not difficult | Slightly difficult | Moderately difficult | Quite difficult | Severely difficult |
| 10. Difficult to use the toilet | Not difficult | Slightly difficult | Moderately difficult | Quite difficult | Severely difficult |
| 11. Difficult to wash body in bath | Not difficult | Slightly difficult | Moderately difficult | Quite difficult | Severely difficult |
| 12. Difficult to go up and down stairs | Not difficult | Slightly difficult | Moderately difficult | Quite difficult | Severely difficult |
| 13. Difficult to walk briskly | Not difficult | Slightly difficult | Moderately difficult | Quite difficult | Severely difficult |
| 14. Difficult to keep oneself neat | Not difficult | Slightly difficult | Moderately difficult | Quite difficult | Severely difficult |
| 15. Difficult to keep walking without rest | 2-3 kilometers or more | About 1 kilometer | About 300 meters | About 100 meters | About 10 meters |
| 16. Difficult to go out to visit neighbors | Not difficult | Slightly difficult | Moderately difficult | Quite difficult | Severely difficult |
| 17. Difficult to carry objects weighing 2 kg | Not difficult | Slightly difficult | Moderately difficult | Quite difficult | Severely difficult |
| 18. Difficult to go out using public transportation | Not difficult | Slightly difficult | Moderately difficult | Quite difficult | Severely difficult |
| 19. Difficult to carry out simple tasks and housework | Not difficult | Slightly difficult | Moderately difficult | Quite difficult | Severely difficult |
| 20. Difficult to carry out load-bearing tasks and housework | Not difficult | Slightly difficult | Moderately difficult | Quite difficult | Severely difficult |
| 21. Difficult to perform sports activities | Not difficult | Slightly difficult | Moderately difficult | Quite difficult | Severely difficult |
| 22. Restricted from meeting friends | Not holding back | Slightly holding back | Moderately holding back | Quite holding back | Completely holding back |
| 23. Restricted from joining social activities | Not holding back | Slightly holding back | Moderately holding back | Quite holding back | Completely holding back |
| 24. Anxious about falls in house | Not anxious | Slightly anxious | Moderately anxious | Quite anxious | Severely anxious |
| 25. Anxious about being unable to walk in the future | Not anxious | Slightly anxious | Moderately anxious | Quite anxious | Severely anxious |
| The severity of locomotive syndrome (LS) is categorized based on the GLFS-25 scores: GLFS-25 <7 points correspond to Stage 0; 7–15 points correspond to Stage 1; 16–23 points correspond to Stage 2 (ie, LS), and ≥24 points correspond to Stage 3. | | | | | |

| **Supplementary Table 2.**  Demographics and clinical characteristics of patients by sex. | | | | |
| --- | --- | --- | --- | --- |
| Variables |  | Male (N=167) | Female (N=424) | P-value |
| Age, yrs | Mean (SD) | 68.9 (12.2) | 66.8 (14.5) | 0.100 |
| Duration of disease, yrs | Mean (SD) | 8.7 (6.4) | 13.0 (10.6) | <0.001 |
| BMI, kg/m^2^ | Mean (SD) | 22.9 (3.4) | 21.6 (3.8) | <0.001 |
| Steinbrocker stage (1/2/3/4), % |  | 43.6/30.7/16.6/9.2 | 36.5/48.8/12.5/3.1 | <0.001 |
| Glucocorticoid use, % |  | 31.7 | 31.4 | 0.922 |
| Methotrexate use, % |  | 44.3 | 65.6 | <0.001 |
| Other csDMARD use, % |  | 49.1 | 42.7 | 0.169 |
| bDMARD or tsDMARD use, % |  | 32.9 | 39.2 | 0.186 |
| Rheumatoid factor positive, % |  | 67.7 | 72.1 | 0.310 |
| CRP, mg/dL | Mean (SD) | 0.5 (0.9) | 0.4 (1.2) | 0.609 |
| MMP-3, ng/mL | Mean (SD) | 127.8 (102.7) | 86.2 (98.4) | <0.001 |
| Swollen joint count | Mean (SD) | 0.4 (1.5) | 0.8 (2.0) | 0.056 |
| Tender joint count | Mean (SD) | 1.9 (3.7) | 2.1 (3.8) | 0.483 |
| Subject's assessment of pain VAS, mm | Mean (SD) | 19.8 (23.5) | 20.5 (23.5) | 0.747 |
| Subject's global assessment of disease activity VAS, mm | Mean (SD) | 20.3 (23.9) | 20.8 (22.9) | 0.804 |
| Physician's global assessment of disease activity VAS, mm | Mean (SD) | 16.2 (21.1) | 17.7 (20.4) | 0.405 |
| CDAI | Mean (SD) | 5.9 (7.2) | 6.7 (7.7) | 0.257 |
| HAQ-DI | Mean (SD) | 0.38 (0.62) | 0.54 (0.76) | 0.013 |
| GLFS-25 | Mean (SD) | 17.5 (18.9) | 20.5 (20.2) | 0.092 |
| LS, % |  | 35.3 | 43.6 | 0.078 |
| Dominant side (right/left/equal), % |  | 53.3/34.1/12.6 | 45.0/35.6/19.3 | 0.083 |
| Grip strength, kg | Mean (SD) | 27.2 (10.5) | 17.4 (7.4) | <0.001 |
| Finger and wrist joint inflammation, % |  | 31.7 | 35.4 | 0.442 |
| See Table 1 for descriptions of abbreviations. | | | | |
